# Neurosteroid Binding and Actions on GABA_A_ Receptors

**DOI:** 10.14789/jmj.JMJ24-0002-R

**Published:** 2024-05-24

**Authors:** YUSUKE SUGASAWA

**Affiliations:** 1 Department of Anesthesiology and Pain Medicine, Juntendo University Faculty of Medicine and Graduate School of Medicine, Tokyo, Japan; 1 Department of Anesthesiology and Pain Medicine, Juntendo University Faculty of Medicine and Graduate School of Medicine, Tokyo, Japan

**Keywords:** neurosteroid, GABA-A receptor, photo-affinity labeling, electrophysiology, binding site

## Abstract

Neurosteroids positively modulate GABA_A_ receptor (GABA_A_R) channel activity by binding to a transmembrane domain intersubunit site. Using photo-affinity labeling and an ELIC-α_1_GABA_A_R chimera, we investigated the impact of mutations within the intersubunit site on neurosteroid binding. These mutations reduce neither photolabeling within the intersubunit site nor competitive prevention of labeling by allopregnanolone. Instead, these mutations change the orientation of neurosteroid photolabeling. The data indicate that mutations at Gln242 or Trp246 that eliminate neurosteroid effects do not eliminate neurosteroid binding within the intersubunit site, but significantly alter the preferred orientation of the neurosteroid within the site. The interactions formed by Gln242 and Trp246 within this pocket play a vital role in determining the orientation of the neurosteroid. We also examined how site-specific binding to three identified neurosteroid-binding sites in the α_1_β_3_GABA_A_R contributes to neurosteroid allosteric modulation. We found that the potentiating neurosteroid, allopregnanolone, but not its inhibitory 3β-epimer epi-allopregnanolone, binds to the canonical β_3_(+)-α_1_(-) intersubunit site that mediates receptor activation by neurosteroids. In contrast, both allopregnanolone and epi-allopregnanolone bind to intrasubunit sites in the β_3_ subunit, promoting receptor desensitization and the α_1_ subunit promoting effects that vary between neurosteroids. Two neurosteroid analogues with diazirine moieties replacing the 3-hydroxyl bind to all three sites, but do not potentiate GABA_A_R currents. One is a desensitizing agent, whereas the other is devoid of allosteric activity. Collectively, these data show that differential occupancy and efficacy at three discrete neurosteroid-binding sites determine whether a neurosteroid has potentiating, inhibitory, or competitive antagonist activity on GABA_A_R.

Steroid hormones can cross the blood-brain barrier and function at the genomic level to produce changes in mood and behavior. These effects develop relatively slowly over minutes to hours, and can persist long after the disappearance of the steroid from the brain. However, certain steroids can produce immediate changes within seconds in neuronal excitability on a timescale that precludes a genomic locus of action.

## What are neurosteroids and where are they made?

**Figure 1:**
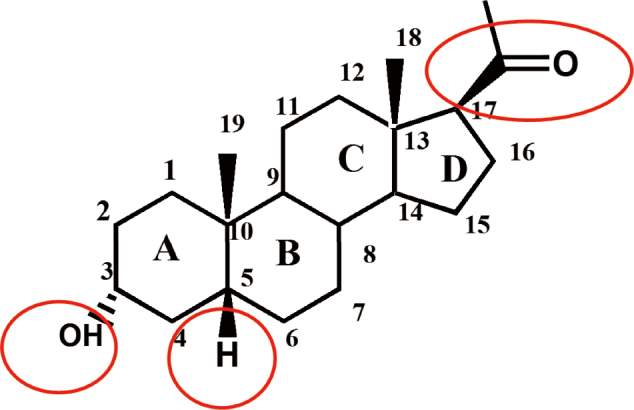
Steroid structure Structure of neurosteroid with carbon atoms numbered and steroid rings labeled. Red circles emphasize the chiral centers at C3, C5 and C17.

The central depressant action of cholesterol was first reported more than three-quarters of a century ago. Subsequently, in the 1940s, it was shown that certain pregnane steroids was able to induce rapid sedation and anesthesia. The steroid structure (Figure 1) contains the chiral centers at C3, C5 and C17 and 3α-hydroxypregnane steroids have been suggested to potentiate γ-aminobutyric acid type A receptors (GABA_A_R).

Steroids were thought to act exclusively as hormones and to originate from endocrine glands, such as the ovaries and adrenal glands, so they would need to cross the blood-brain barrier before they could influence neuronal signaling. However, the brain is also a steroidogenic organ. Certain neurons and glia in the central nervous system express the enzymes that are required for the local synthesis of pregnane neurosteroids (NS)^1)^.

## What do endogenous neurosteroids do?

GABA is released from vesicles rapidly activates a family of postsynaptic GABA_A_R, which gives rise to inhibitory postsynaptic current. NS that are released locally from neurons or glia prolong the decay of such responses, enhancing synaptic inhibition. In addition, certain neurons contain extrasynaptically-located receptors that are activated by low levels of ambient GABA to cause a ‘tonic’ inhibition. Some NS appear to engage other targets, such as NMDA receptors, T-type calcium channels and toll-like receptors^2)^.

## How do neurosteroids work?

In the early 1980s, Harrison and Simmonds showed that certain endogenous steroids are potent positive allosteric modulators of the GABA_A_R^3)^. Subsequent studies also showed potent stereo-selective GABA-modulatory effect of NS.

The GABA_A_R are pentameric ligand-gated ion channels that include other receptors such as acetylcholine receptors, glycine receptors and serotonin receptors. Transmembrane domains of GABA_A_R are one of main targets of anesthetic action. NS are thought to potentiate the GABA_A_R by direct interactions at specific sites within the transmembrane domains. The second part of four transmembrane domains from each subunit construct an ion channel pore of the GABA_A_R. Recent photo- affinity labeling and crystallographic studies have shown that NS bind to a specific site between adjacent subunits that mediates GABA_A_R potentiation^4-8)^.

## Identifying neurosteroid anesthetic binding sites

**Figure 2:**
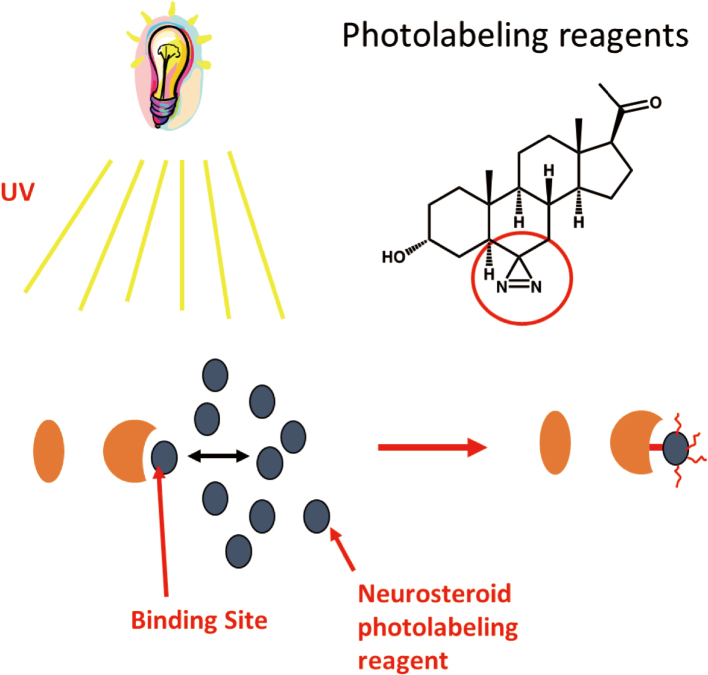
Principle of photolabeling A neurosteroid photolabeling reagent covalently attaches to a binding site with UV irradiation.

My research focuses on identifying NS anesthetic binding sites on membrane proteins by means of photo-affinity labeling, mass spectrometry and cryogenic electron microscopy. Photoaffinity labeling is a rapid and inexpensive method. It can be performed in cells or native membranes. However, there are a few limitations of photo-ligands in terms of specificity, efficiency, and non-identity to anesthetic. Lack of 3-D structure is also a limitation of photoaffinity labeling. The principle of photolabeling is that a NS photolabeling reagent covalently attaches to a binding site with UV irradiation (Figure 2). Mass spectrometric analysis on photolabeled membrane protein can localize binding sites, stoichiometry and photolabeling efficiency.

With this novel approach, we have identified NS binding sites in GABA_A_R transmembrane domains. One is between β_3_ and α_1_ subunits, others locate within a β_3_ or α_1_ subunit. We call them as inter-subunit binding site and intra-subunit binding site, respectively. We have shown that both inter- and α_1_ intra-subunit sites contribute to NS potentiation of GABA_A_R by electrophysiological experiments and site-directed mutagenesis^7)^.

## Neurosteroid hydrogen bonding drives binding orientation

**Figure 3:**
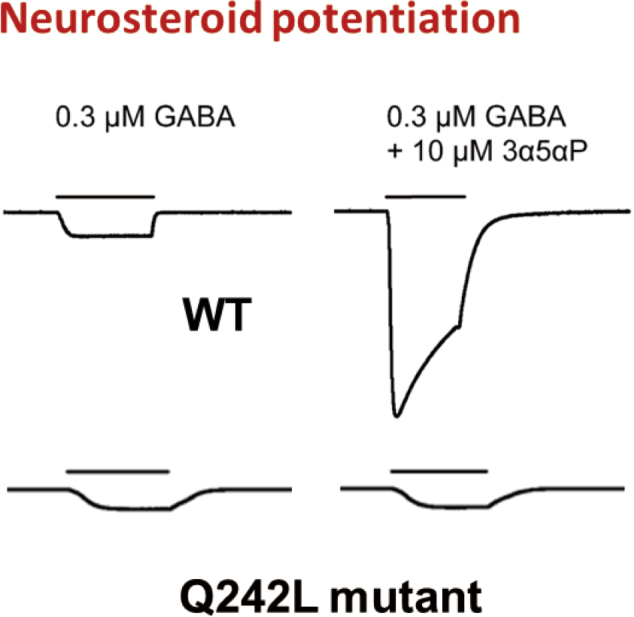
Neurosteroid potentiation Sample current traces from α_1_β_3_ GABA_A_R WT activated by 0.3 µM GABA showing potentiation by 10 µM 3α5αP and the absence of potentiation by 10 µM 3α5αP in the mutant, α_1_(Q242L)β_3_ GABA_A_R activated by 0.3 µM GABA.

Within the intersubunit site, two key residues, Gln242 and Trp246, are shown to form hydrogen bond and hydrophobic ring-stacking interactions, respectively, with the NS. To date, functional studies have demonstrated that mutations of the two key residues abolish the NS potentiating effect (Figure 3). Based on these findings, it was suggested that these mutations abolish potentiation by reducing or eliminating NS binding. There was, however, no experimental evidence to evaluate whether these mutations alter NS binding or affect transduction of the binding signal. We therefore tested the effect of mutations on the efficiency, stoichiometry and sites of NS photolabeling in order to evaluate whether these mutations reduce NS binding, change binding orientation or affect transduction of the binding signal. To enable the use of mass spectrometric analysis of photolabeled GABA_A_R transmembrane domains and the expression of multiple mutants, we expressed an ELIC-α_1_GABA_A_R chimera.

Our NS photolabeling reagents labeled the ELIC-α_1_GABA_A_R chimera with stoichiometry of two using two established allopregnanolone (3α5αP)-based photolabeling reagents, KK123 and KK200. The primary aim of this study is to test whether the loss of these functionally important interactions disrupt NS binding. To address this, we photolabeled ELIC-α_1_GABA_A_R chimera mutants with KK200. Analysis of the labeled mutants by intact protein mass spectrometry showed labeling efficiencies identical to WT, suggesting that NS still bind to mutant ELIC-α_1_GABA_A_R. This finding contradicts the idea that these mutations disrupt NS binding. To confirm that the endogenous NS, 3α5αP, also binds to the intersubunit site of the Q242W or W246L mutant transmembrane domains, we tested whether 100 μM 3α5αP competitively reduces the labeling efficiency of 10 μM KK200 in WT, Q242W and W246L mutants. Indeed, 3α5αP reduced the photolabeling efficiency of KK200 for the intersubunit site to the same extent in WT and both mutants suggesting that 3α5αP binds to the intersubunit site in both WT and the mutants.

Next, we used a middle-down mass spectrometric analysis to identify the residues labeled by KK200 in each mutant. Just as in WT, KK200 labeled the same Asn408 in the intrasubunit site in all three mutants. Surprisingly, unlike WT, where KK200 labeled Tyr309 in TM3 which forms the intracellular end of the intersubunit pocket, KK200 labeled Phe298 in the mutants. Thus, KK200 photolabeling of Phe298 in all three mutants, instead of Tyr309, indicates that Gln242 and Trp246 are important determinants of NS binding orientation.

It is possible that the changes in photolabeling observed for KK200 are not reflective of binding for the endogenous NS, 3α5αP. To address this possibility, we examined computational docking of 3α5αP to the WT and Q242W α_1_GABA_A_R transmembrane domains. The respective binding modes of the docking results are consistent with KK200 labeling of Tyr309 in WT and Phe298 in the Q242W mutant. Collectively, the data indicate that mutations at Gln242 or Trp246 that eliminate NS effects do not eliminate NS binding within the intersubunit site, but significantly alter the preferred orientation of the NS within the site^8)^.

## Inter- and α_1_ intra-subunit sites contribute to neurosteroid potentiation of GABA_A_ receptors

Our recent photo-labeling studies have confirmed that there are multiple positive allosteric modulatory (PAM)-NS-binding sites on α_1_β_3_ GABA_A_R^7)^. In addition to the site at the interface between the transmembrane domains of adjacent subunits which is called an intersubunit site, we identified NS binding sites within the α-helical bundles of both the α_1_ and β_3_ subunits of α_1_β_3_ GABA_A_R. 3α5αP binds to all three sites, and mutagenesis of these sites suggests that the intersubunit and α_1_ intrasubunit sites, but not the β_3_ intrasubunit site, contribute to PAM activity of 3α5αP. A functional effect for NS binding to the β_3_ intrasubunit site had not been identified.

## Dissociation of neurosteroid GABA_A_ receptor activation and enhancement of orthosteric ligand binding

We hypothesized that various NS analogues preferentially bind to one or more of the three NS binding sites in the α_1_β_3_ GABA_A_R, stabilizing distinct conformational states (i.e. resting, open or desensitized). To achieve this goal, we used two endogenous NS, the PAM-NS 3α5αP and the negative allosteric modulatory (NAM)-NS epi-allopregnanolone (3β5αP) and two NS analogues, KK148 and KK150, in which a diazirine replaced the function-critical 3-OH group. We examined site-specific NS binding and effects using photolabeling and measurements of channel gating and orthosteric ligand binding. The NS lacking a 3α-OH were devoid of PAM activity, but surprisingly, KK148 and 3β5αP enhanced the affinity of orthosteric ligand, [^3^H]muscimol binding. We interpret this finding as evidence that these compounds preferentially bind to and stabilize desensitized receptors, since both open and desensitized GABA_A_R exhibit enhanced orthosteric ligand-binding affinity^9)^.

## Effects of neurosteroids on desensitization of GABA_A_ receptor

Many 3β-OH NS are GABA_A_R NAM^10)^. Co-application of 3β5αP with 1 mM GABA preferentially inhibited steady-state rather than peak currents. The inhibitory effect of 3β5αP was not observed in receptors with the α_1_(V256S) TM2 pore-lining mutation, which was previously shown to remove the inhibitory effects of steroids^10, 11^^)^. To examine the inhibitory effect of the NS analogues, we activated α_1_β_3_ GABA_A_R with a saturating concentration of GABA and tested the effect of the NS on steady-state currents. KK148 and 3β5αP both decreased steady-state currents, whereas KK150 did not.

## Which sites do neurosteroid analogues bind?

To determine whether KK148 and 3β5αP stabilize a desensitized conformation of the GABA_A_R by selectively binding to one or more of the identified NS binding sites on the GABA_A_R, we first determined which of the identified NS-sites they bind. We have previously shown that the 3α5αP-analogue photolabeling reagent, KK200 labels the β_3_(+)–α_1_(-) intersubunit and a α_1_ intrasubunit sites on α_1_β_3_ GABA_A_R, and that photolabeling can be prevented by a 10-fold excess of 3α5αP. As a first step to determine the binding sites for 3β5αP, KK148 or KK150, we examined whether a 10-fold excess of these compounds (30 mM) prevented KK200 (3 mM) photolabeling of either binding site.

KK148, KK150, 3α5αP and 3β5αP all prevented KK200 photolabeling of α_1_Asn408 in the α_1_ intrasubunit site, consistent with their binding to this site. In contrast, KK148, KK150 and 3α5αP but not 3β5αP prevented labeling of the intersubunit site, indicating that 3β5αP does not bind to the intersubunit site.

The KK148- and KK150-photolabeled samples were also analyzed to directly identify the sites of adduction. In both the KK148- and KK150-labeled samples, photolabeled peptides were identified from the TM4 helices of both the α_1_ and β_3_ subunits.

We have also shown that KK123 labeling of the α_1_ intrasubunit (α_1_Tyr415) and β_3_ intrasubunit (β_3_Tyr442) sites can be prevented by a 10-fold excess of 3α5αP. Thus, we examined whether 3β5αP inhibited photolabeling by KK123. 3β5αP completely inhibited KK123 photolabeling at both intrasubunit sites.

Collectively, the data show that KK148, KK150 and 3α5αP bind to all three of the identified NS binding sites. In contrast, 3β5αP selectively binds to the two intrasubunit binding sites, but not to the β_3_(+)–α_1_(-) intersubunit site^12)^.

## Sites of neurosteroid action on [^3^H]muscimol binding

To determine which of the previously identified binding sites contributes to NS enhancement of [^3^H]muscimol binding, we performed site-directed mutagenesis of the NS-binding sites previously determined by photolabeling. Specifically, α_1_(Q242L)β_3_ targets the β_3_(+)–α_1_(-) intersubunit site, α_1_(N408A/Y411F)β_3_ and α_1_(V227W)β_3_ the α_1_ intrasubunit site, and α_1_β_3_(Y284F) the β_3_ intrasubunit site.

Mutations in the β_3_(+)–α_1_(-) intersubunit and α_1_ intrasubunit sites decreased 3α5αP enhancement of [^3^H]muscimol binding by ~80%, while mutation of the β_3_ intrasubunit site led to a small decrease. The residual enhancement of [^3^H]muscimol binding observed in receptors with mutations in the intersubunit or α_1_ intrasubunit site occurs at 10-fold higher concentrations of 3α5αP than WT and receptors with mutations in the β_3_ intrasubunit site, suggesting that 3α5αP binds to the β_3_ intrasubunit site with lower affinity. In contrast, mutations in the α_1_ and β_3_ intrasubunit sites, but not the intersubunit site decreased the enhancement of [_3_H]muscimol binding by 3β5αP and KK148.

Collectively, these results show that multiple NS binding sites contribute to enhancement of [^3^H]muscimol affinity and that potentiating NS (3α5αP) and non-potentiating NS (3β5αP, KK148 and KK150) have both common and distinct sites of action^12)^.

## Sites of 3β5αP desensitization of GABA_A_ receptor

To further explore the relationship between desensitization and enhancement of [^3^H]muscimol binding, we examined the consequences of mutations to these sites on physiological measurements of desensitization induced by NS. 3β5αP reduced the steady-state current by 23.0%. Mutations in the α_1_ and β_3_ intrasubunit sites prevented 3β5αP-enhanced desensitization by ~67%, whereas mutation in the β_3_(+)–α_1_(-) intersubunit site was without effect. Receptors with mutations in both the α_1_ and β_3_ intrasubunit sites showed less NS-enhancement of desensitization than receptors with mutations in either of the intrasubunit sites alone, indicating that both intrasubunit sites contribute to the desensitizing effect. Since mutations of the α_1_ and β_3_ intrasubunit sites also disrupt 3β5αP-enhancement of [^3^H]muscimol binding, we conclude that 3β5αP binding to these intrasubunit sites stabilizes the desensitized state of the GABA_A_R and enhances [^3^H]muscimol binding.

## Sites of 3α5αP desensitization of GABA_A_ receptor

3α5αP binds to all three of the neurosteroid binding sites on α_1_β_3_ GABA_A_R, and mutations in all three sites reduce 3α5αP enhancement of [^3^H]muscimol binding. This suggests the possibility that activation by 3α5αP (mediated primarily by the β_3_(+)–α_1_(-) intersubunit site) masks a desensitizing effect mediated through the β_3_ and/or α_1_ intrasubunit binding sites. To determine whether intrasubunit binding sites mediate increased desensitization by 3α5αP, we examined the effect of 3α5αP on steady-state currents in receptors with mutations in the α_1_ or β_3_ intrasubunit site. Mutations in the intrasubunit sites were prepared with a background α_1_(Q242L)β_3_ mutation to remove 3α5αP activation and focus on the effects of 3α5αP on the equilibrium between the open and desensitized states. 3α5αP produced a small reduction in steady-state current in α_1_(Q242L)β_3_ receptors with mutations in neither of the intrasubunit sites. This inhibitory effect was eliminated by α_1_(V256S)β_3_, indicating that it was due to receptor desensitization. In receptors with combined mutations in the intersubunit and α1 intrasubunit sites, 3α5αP significantly inhibited the steady-state current, an effect that was markedly reduced by mutations in the β_3_ intrasubunit site. These data suggest that 3α5αP exerts a desensitizing effect by binding to the β_3_ intrasubunit site and that 3α5αP binding to the α_1_ intrasubunit site does not promote desensitization.

## Conclusion

Here, we examined how site-specific binding to the three identified neurosteroid sites on α_1_β_3_ GABA_A_R contributes to the PAM vs. NAM activity of epimeric 3-OH NS. We found that the PAM-NS 3α5αP, but not the NAM-NS 3β5αP, binds to the canonical β_3_(+)–α_1_(-) intersubunit site that mediates receptor potentiation, explaining the absence of 3β5αP PAM activity. In contrast, 3β5αP binds to intrasubunit sites in the α_1_ and β_3_ subunits, promoting receptor desensitization. Two synthetic NS with diazirine moieties at C3 (KK148 and KK150) were used to identify NS binding sites and shown to bind to the intersubunit as well as both intrasubunit sites. KK148 is an efficacious desensitizing agent, acting through the α_1_ and β_3_ intrasubunit NS binding sites. KK150, the 17α-epimer of KK148, binds to all three NS binding sites, but neither activates nor desensitizes GABA_A_R, suggesting a potential chemical scaffold for a general NS antagonist.

Collectively, these data show that differential occupancy of and efficacy at three discrete NS binding sites determines whether a NS ligand has PAM, NAM, or potentially NS antagonist activity on GABA_A_R.

It remains to be determined whether there are additional unique sites on γ or δ subunits or isoform specificity in neurosteroid binding within the α_1__-__6_ or β_1__-__3_ subunits. Either of these possibilities could provide specific pharmacologic targets for GABA_A_R subtypes.

## Funding

No funding was received.

## Author contributions

The author read and approved the final manuscript.

## Conflicts of interest statement

The author declares that there is no conflict of interest.
